# The Effect of Autologous Adipose-Derived Stromal Vascular Fractions on Cartilage Regeneration Was Quantitatively Evaluated Based on the 3D-FS-SPGR Sequence: A Clinical Trial Study

**DOI:** 10.1155/2022/2777568

**Published:** 2022-01-25

**Authors:** Yin Zhang, Qing Bi, Junchao Luo, Yu Tong, Taihen Yu, Qiong Zhang

**Affiliations:** ^1^Department of Orthopedic Surgery, Zhejiang Provincial People's Hospital and People's Hospital of Hangzhou Medical College, No. 158 Shangtang Road, Hangzhou, 310014 Zhejiang, China; ^2^The First Affiliated Hospital of Bengbu Medical University, Bengbu, Anhui 233004, China; ^3^Department of Operating Room, Zhejiang Provincial People's Hospital and People's Hospital of Hangzhou Medical College, No. 158 Shangtang Road, Hangzhou, 310014 Zhejiang, China

## Abstract

**Background:**

Numerous reports confirmed the safety and clinical efficacy of autologous adipose-derived stromal vascular fractions (SVF), which have recently been used to treat osteoarthritis (OA). However, there is still no consensus as to whether SVF can promote cartilage regeneration. Herein, the purpose of our study was to evaluate the effectiveness of SVF versus hyaluronic acid (HA) in cartilage regeneration by establishing a cartilage model based on the three-dimensional fat-suppressed spoiled gradient recalled echo (3D-FS-SPGR) sequence.

**Methods:**

Patients with symptomatic OA were recruited in our research, who were randomized into two groups. Meanwhile, patients in Kellgren-Lawrence (K-L) grades 2 and 3 were distinguished in each group. In the test group, patients received SVF injections of the knee, while patients in the control group received the same dose of HA. Each patient underwent the 3D-FS-SPGR sequence to establish a cartilage model at baseline, 6 months, and 12 months, respectively. The cartilage was characterized into six regions, and relevant parameters of the cartilage model were counted. Clinical and radiographic scores were recorded in one-year follow-up.

**Results:**

In all regions, the thickness and volume of cartilage defect and the volume of healthy cartilage were improved to some extent in the test group, especially the medial femoral condyle (MF) and medial tibial condyle (MT). In grades 2 and 3, the thickness and volume of cartilage defect decreased by 0.92 ± 0.18 mm and 1.03 ± 0.23 mm and 84.00 ± 32.30 mm^3^ and 130.30 ± 49.56 mm^3^ in MF and by 0.96 ± 0.22 mm and 0.99 ± 0.14 mm and 64.18 ± 21.40 mm^3^ and 95.11 ± 19.93 mm^3^ in MT, respectively. No such phenomenon was observed in the control group. Meanwhile, the SVF-treated knees showed significant improvement in clinical and radiographic scores at 12 months. Nevertheless, these scores of the control group became worse at 12-month follow-up visit.

**Conclusion:**

Taken together, this study shows that intra-articular injection of SVF markedly improved the clinical symptoms without adverse events, thereby repairing the damaged articular cartilage through cartilage regeneration.

## 1. Background

Osteoarthritis (OA) is a common chronic disease of the joints, which is characterized by osteophyte formation, changes to the subchondral bone, degeneration of ligaments and menisci, pain, stiffness, and loss of joint function [1, 2]. Several studies have established that knee OA is a highly prevalent form of arthritis that contributes to arthralgia and disability, especially in elderly people [3].

To date, more than 50 therapies of pharmacological, nonpharmacological, and surgical approach have been documented by scholars. Intra-articular injection of hyaluronic acid (HA) is effective in improving symptoms and slowing the progression of OA, but they do not mention the degeneration and regeneration of articular cartilage [4]. Therefore, most patients cannot inevitably avoid taking the road of total knee arthroplasty (TKA) in the end [5, 6]. In this respect, it is therefore of great significance to find a new and effective therapy for alleviating the clinical symptoms of OA and preventing the degeneration of articular cartilage.

Since the discovery of the multipotent stem cell population in adipose tissue by Zuk et al., cell-based regenerative therapy has gradually become a possible method for cartilage regeneration [7]. Recent related studies have also confirmed that mesenchymal stem cells (MSCs) and adipose-derived stem cells (ADSCs) possess the potential to differentiate into chondrocytes. However, MSCs and ADSCs need to take several weeks in specialized laboratory for cell isolation and expansion, which will increase the economic burden of the patients [8, 9]. Some scholars have proposed a more effective method to collect and manage ADSCs using stromal vascular fraction (SVF) [10]. Furthermore, adipose-derived SVF comprises numerous regenerative cells, such as ADSCs, blood cells, pericytes, fibroblasts, macrophages, smooth muscle cells, endothelial cells, and their precursors. It also exhibits the benefits of easy isolation and use without culturing or differentiation, consequently responding to the local environment of OA by some inflammatory factors [11–13]. Multiple recent reports have proven that the use of intra-articular SVF injections can effectively relieve the clinical symptoms of patients [14–17]. Nonetheless, despite these intriguing results, it remains unclear whether the SVF injections can promote regeneration of the articular cartilage, requiring further exploration. Inconsistent findings have been reported regarding this topic, whereby some studies reported evidence of cartilage tissue regeneration, while others claimed that no change is observed [18–21].

We thus designed a clinical trial about autologous adipose-derived SVF versus HA in the treatment of patients with knee OA Kellgren-Lawrence (K-L) grades 2 and 3 [22]. This study sought to establish a three-dimensional (3D) cartilage model by using a special sequence to quantitatively examine the effect of SVF and HA on cartilage regeneration.

## 2. Materials and Methods

### 2.1. Patients and Study Design

The trial was registered at the Chinese Clinical Trial Registry (ChiCTR2100042930). All experimental protocols used in this study were approved by the Ethics Committees of Zhejiang Provincial People's Hospital. Patients enrolled in this study provided signed written informed consent. This was a prospective double-blinded randomized study conducted at a single center. Eligible patients included were aged between 18 and 70 years, with OA K-L grades 2 and 3, exhibiting substantial pain and loss of function, failure of conservative therapy, and had an initial pain evaluated at four or greater on a ten-point visual analog scale (VAS) in the knee joint. On the other hand, exclusion criteria are comprised of secondary arthritis (for example, secondary knee OA, rheumatoid arthritis, gouty arthritis, and previous articular fractures), having problem with anesthesia (according to the American Society of Anesthesiologists score), contraindicating MRI examination, other causes of knee pain such as diffuse edema, meniscus tear, and others, a history of liposarcoma and other cancers, intra-articular injection of hyaluronic acid or other drugs in the preceding 3 months, end-stage OA, patients with recent surgery, abdominal hernia, and coagulopathy.

The complete randomization process was accomplished by an assistant accountant who was blinded to the patients' data using SPSS 20.0 software (version 20.0, IBM Corporation, NY, US). First, we listed 1–100 serial numbers (patient serial number) in accordance with the outpatient order. Second, 100 random numbers were generated by Rv.Uniform (0, 1) and matched number by number with 100 patients' serial numbers. Finally, the 100 random numbers were arrayed in ascending order; the corresponding patients of the first fifty random numbers were injected with 4 ml SVF and 4 ml hyaluronic acid (SOFAST, Freda, china) in the last 50 random numbers.

To evaluate the grade of OA, an initial X-ray image was used following the K-L criteria, and subsequently, patients belonging to grades 2 and 3 were selected. Afterward, patients who underwent MRI included conventional and three-dimensional fat-suppressed spoiled gradient recalled echo (3D-FS-SPGR) sequences; the radiologist is not informed of the patient's treatment. According to the conventional sequence, the whole-organ magnetic resonance imaging score (WORMS) was recorded to evaluate the knee, and magnetic resonance observation of cartilage repair tissue (MOCART) was recorded to assess the cartilage repair tissue. While the 3D-FS-SPGR sequence was employed to build the 3D cartilage model and measure the related parameters, the visual analog scale (VAS) and Western Ontario and McMaster Universities Osteoarthritis Index (WOMAC) questionnaire were used to evaluate the pain and function of the patient. We also examined the range of motion (ROM) during the follow-up period.

### 2.2. Establishment of the 3D Cartilage Model

The MRI scanning was performed on a clinical 3.0 T system (GE Healthcare, Waukesha, WI, USA), including the 3D-FS-SPGR and conventional sequence (TE: 34.5 ms; TR: 2000 ms; the number of excitations: 2; FOV: 16 × 16 cm; slice thickness: 4 mm; interslice gaps: 5 mm; coil: knee coil; the total scan time: 180 s; acquired slices: 21 slices; and flip angle: 0°). Using the 3D-FS-SPGR sequence, each patient was examined before SVF injection. Acquisition parameters for the 3D-FS-SPGR sequence were as follows: TE: 3 ms; TR: 14.6 ms; acquisition matrix: 512 × 512; the number of excitations: 2; FOV: 16 × 16 cm; slice thickness: 0.6 mm; interslice gaps: 0 mm; receiver BW: ±41.7 kHz; coil: knee coil; total scan time: 1220 s; acquired slices: 276 contiguous slices; flip angle: 0°; and plane resolution: 0.60 mm × 0.60 mm [23].

**Figure 1:**
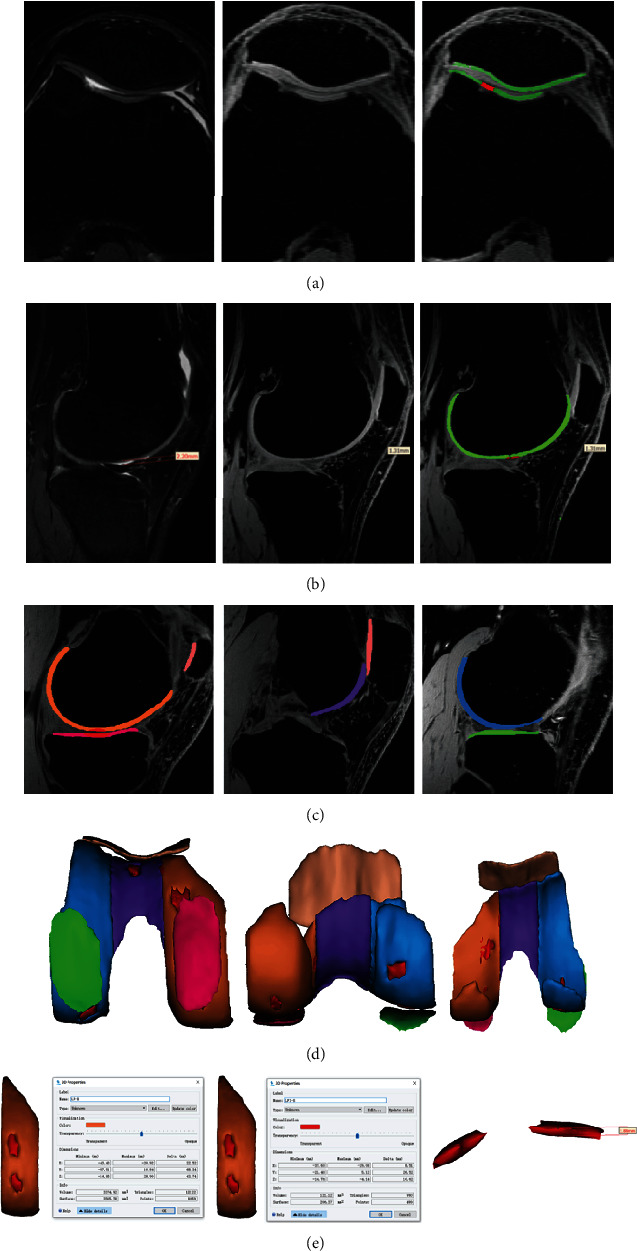
The process of establishing the cartilage model. Different color masks used to distinguish healthy cartilage from cartilage defects by setting the threshold are shown. Illustrating the injury of the whole layer of cartilage and partial cartilage defects (a, b). The cartilage of the knee joint was divided into six regions with different color masks, namely, lateral femoral condyle (LF), femoral intercondylar (T), medial femoral condyle (MF), lateral tibia condyle (LT), medial tibia condyle (MT), and patella (P), and the knee cartilage model was established (c, d). The parameters of the model were measured (e) (for example, in the lateral femoral condyle, the thickness, volume, and surface of cartilage defect were 1.88 mm, 121.12 mm^3^, and 206.37 mm^2^, respectively. The volume of healthy cartilage was 3374.92 mm^3^).

To build the 3D cartilage model, the original data of the 3D-FS-SPGR sequence was converted to Digital Imaging and Communications in Medicine (DICOM) format and transferred into the Mimics 20.0 software (Materialise, Leuven, Belgium). First, all layers of cartilage defects were detected using 3D-FS-SPGR and conventional sequences. An appropriate segmentation threshold (1849-3445 GV, the segmentation threshold was determined by the cartilage to be segmented) was set for retaining the healthy cartilage of the knee joint, saving the results as the green mask. Following this, the cartilage defect was segmented by another mask, then saving it as a red mask. The healthy cartilage and cartilage defects of the knee joint are segmented by the use of different masks. After the layer-by-layer hierarchical image processing, the cartilage model was characterized into six regions, namely, medial femoral condyle (MF), lateral femoral condyle (LF), femoral intercondylar (T), medial tibia condyle (MT), lateral tibia condyle (LT), and patella (P) [24]. Different color masks represented different areas, while cartilage defects were represented by red masks (Figures 1(a) and 1(b)). Then, the cartilage tissue for each layer was preserved, the contours of knee cartilage were calculated, and the cartilage model of each region was established (Figures 1(c) and 1(d)). The volume of healthy cartilage, as well as the volume, surface, and thickness of cartilage defects, was measured by the same professional surveyor (Figure 1(e)), and the professional surveyor was unaware of the patient's information. After one week, the cartilage model was reestablished, and the above-mentioned data were measured and averaged.

### 2.3. Clinical and Radiological Evaluation

The VAS and WOMAC questionnaires were used for the evaluation of pain and functional limitation. The WOMAC score includes pain (five items, score range 0-20), stiffness (two items, score range 0-8), and physical function (seventeen items, score range 0-68), with a total score ranging from 0 (best health) to 96 (worst health). The total score of VAS ranged from 0 (best) to 10 (worst). Additionally, we recorded the ROM of the knee joint during the follow-up. Finally, we assessed the safety of SVF and HA by analyzing the incidence rate of adverse events (AE) and serious adverse events (SAE).

In order to minimize the influence of knee joint loading on the results of MRI, patients were required to rest for 30 minutes before examination. We employed the MOCART score to examine the cartilage repair, while the WORMS was used for the assessment of the knee [25, 26].

### 2.4. SVF Isolation and Injection

For this experiment, patients were not allowed to take aspirin, thrombolytic or antiplatelet medication, corticosteroids, and nonsteroidal anti-inflammatory drugs within one week before liposuction. Also, they all fasted for liquids and solids at least six hours before the operation. The operation was performed by the same skilled plastic surgeon who was blinded to patient information. After disinfection of the abdomen, the surgeon made two small incisions around the umbilical cord and obtained 100 to 150 ml of adipose tissue from the subcutaneous tissue using the superwet technique. Briefly, lipoaspirates were washed with phosphate-buffered saline, while the mesh filter was applied to remove containing residual blood cells and tissue fragments. Next, an equal volume of digestive enzyme (type I collagenase with the concentration of 5%; Worthington, Lakewood, NJ, USA) was mixed with the washed adipose tissue and placed in a shaking incubator at 37°C for 30 minutes. The resulting mixture was then centrifuged at a rate of 1000 g for 10 minutes, and subsequently, the supernatant (Eppendorf 5810R, Germany) was discarded. After this, the remnant SVF at the bottom was resuspended in phosphate-buffered saline (PBS) up to a volume of 4.5 ml SVF, whereas an automatic cell counter (Countstar IC1000, China) was used to quantify cell quantity and viability.

In short, about 4 ml of SVF suspension was injected into the region of the cartilage defect by a trained experienced orthopedic surgeon who was blinded to patient information. The patient was supine, the knee joint was straightened, and the intersection of the upper edge of the patella and the outer edges of the patella were the location of injection. The injection was performed diagonally to the center of the patellofemoral joint at an angle of 45°. Upon the injection of SVF, subcuticular suture and pressure dressing were performed. All the operations were performed by the same experienced orthopedic surgeon.

### 2.5. Statistical Analysis

Changes in all follow-up data were determined using a paired *t*-test. The discrete data were analyzed by the chi-square test. The value of *p* < 0.05 was considered statistically significant. Data displayed in the graphs are means with standard deviation. All statistical data analyses were executed using SPSS software (version 20.0, IBM Corporation, NY, US).

## 3. Results

### 3.1. Patient Characteristics and Safety

**Figure 2:**
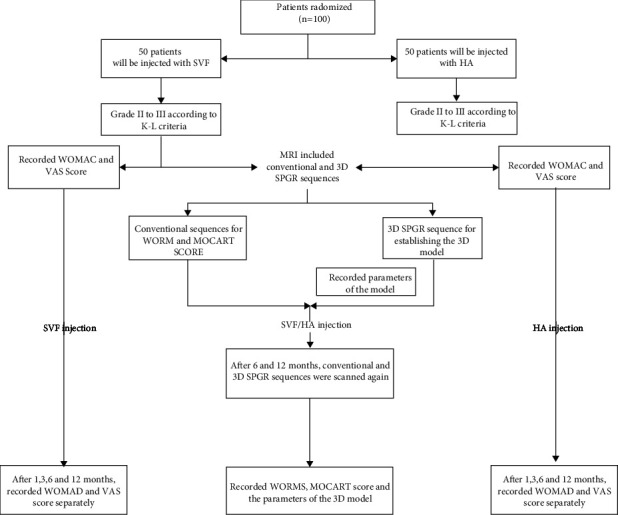
Flowchart of the clinical trial.

From January 2018 to May 2021, the 95 patients who satisfied the standard were divided into two groups (Figure 2). The patients' characteristics showed no significant difference in age, gender distribution, BMI, and K-L grade between the two groups (Table 1). Finally, 47 patients (53 knees) with OA received an intra-articular injection of SVF, and 48 patients (51 knees) received HA. During the follow-up period, no serious AE (infection, allergy, and poor wound healing) happened. The most common AE were pain and swelling of the knee, which occurred in 21 patients (22.11%). After treatment with anti-inflammatory and analgesic drugs, the pain and swelling of all knees were relieved in two weeks. These patients will still be enrolled in clinical trials as long as they do not develop complications such as infections and allergy.

### 3.2. Changes in Parameters of the 3D Cartilage Model

**Figure 3:**
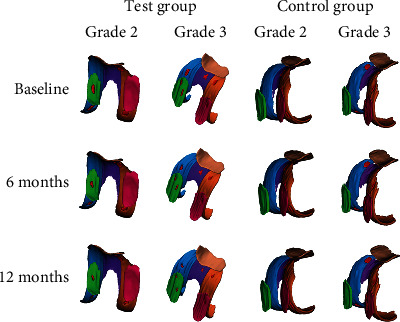
Cartilage model of the SVF- and HA-treated knee established at baseline and 6 and 12 months. The cartilage defect of the SVF-treated knee with K-L grade 2 and 3 OA showed good repair; the cartilage defect of the HA-treated knee with K-L grade 2 and 3 OA showed no improvement.

**Figure 4:**
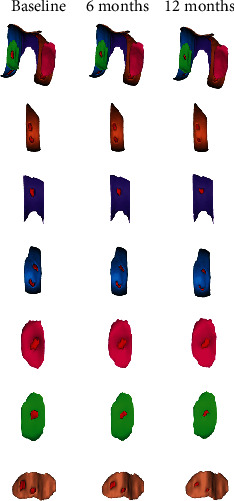
Cartilage model of the SVF-treated knee established at baseline and 6 and 12 months. The cartilage defect of the knee joint with OA K-L grade 2 showed good repair (a). Change of cartilage defects in the LF (b), T (c), MF (d), LT (e), MT (f), and P (g) after injection.

To establish the 3D cartilage model, all patients finished the examination of the 3D-FS-SPGR sequence at baseline and 6 and 12 months (Figure 3). In the test group, the parameters of 3D cartilage model improved in both patients with OA K-L grades 2 and 3 (Table 2). In grade 2, the thickness of cartilage defect decreased from 1.53 ± 0.23 mm to 0.92 ± 0.18 mm in MF (40% decrease; *p* < 0.001); from 1.46 ± 0.30 mm to 1.17 ± 0.26 in LF (20% decrease; *p* < 0.001); from 1.45 ± 0.25 mm to 1.25 ± 0.21 mm in T (14% decrease; *p* < 0.05); from 1.43 ± 0.26 mm to 0.96 ± 0.22 mm in MT (33% decrease; *p* < 0.001); from 1.34 ± 0.19 mm to 1.13 ± 0.18 mm in LT (16% decrease; *p* < 0.001); and from 1.29 ± 0.19 mm to 1.01 ± 0.15 mm in P (22% decrease; *p* < 0.001). The volume of cartilage defect decreased by 84.00 ± 32.30 mm^3^ in MF (52% decrease; *p* < 0.001); by 94.73 ± 45.55 mm^3^ in LF (35% decrease; *p* < 0.001); by 64.18 ± 21.40 mm^3^ in MT (54% decrease; *p* < 0.001); and by 88.66 ± 28.04 mm^3^ in LT (26% decrease; *p* < 0.001), but not in T and P, from 147.91 ± 61.35 mm^3^ to 112.80 ± 56.09 mm^3^ in T (24% decrease; *p* = 0.085) and from 137.29 ± 53.30 mm^3^ to 102.15 ± 43.47 mm^3^ in P (26% decrease; *p* = 0.095). We further found that the surface of cartilage defect decreased in MF, LF, T, MT, and LT, showing a statistically significant difference. Nevertheless, we observed no statistical difference in P (27% decrease; *p* = 0.057). As for the healthy cartilage, we generally identified no significant difference, except for MT and LT. Subsequently, we noted that the volume of healthy cartilage increased from 1647.92 ± 200.24 mm^3^ to 1783.31 ± 202.94 mm^3^ and from 1613.65 ± 147.04 mm^3^ to 1694.24 ± 150.56 mm^3^ in MT (8% increase; *p* < 0.05) and LT (5% increase; *p* < 0.05), respectively (Figure 4).

**Figure 5:**
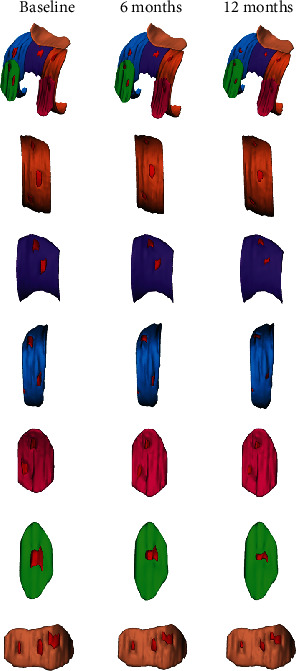
Cartilage model of the SVF-treated knee established at baseline and 6 and 12 months. The cartilage defect of the knee joint with OA K-L grade 3 showed good repair (a). Change of cartilage defects in the LF (b), T (c), MF (d), LT (e), MT (f), and P (g) after injection.

Similar to grade 2 OA, the thickness of cartilage defect reduced in MF, LF, T, MT, LT, and P, indicating a significant difference in grade 3 (Figure 5). The volume of cartilage defect decreased from 278.10 ± 110.58 mm^3^ to 130.30 ± 49.56 mm^3^ in MF (53% decrease; *p* < 0.001); from 229.23 ± 94.05 mm^3^ to 162.17 ± 70.92 mm^3^ in LF (29% decrease; *p* < 0.001); from 196.75 ± 77.85 mm^3^ to 141.78 ± 59.94 mm^3^ in T (28% decrease; *p* < 0.05); from 200.96 ± 48.48 mm^3^ to 95.11 ± 19.93 mm^3^ in MT (53% decrease; *p* < 0.001); from 154.40 ± 48.17 mm^3^ to 110.57 ± 39.8 mm^3^ in LT (28% decrease; *p* < 0.05); and from 140.84 ± 56.97 mm^3^ to 98.75 ± 42.84 mm^3^ in P (30% decrease; *p* < 0.05). The surface of cartilage defect decreased from 525.43 ± 167.38 mm^2^ to 286.18 ± 108.47 mm^2^ and from 410.59 ± 88.53 mm^2^ to 208.12 ± 42.70 mm^2^ in MF (46% decrease; *p* < 0.001) and MT (49% decrease; *p* < 0.001), respectively. We also noted that the volume of healthy cartilage increased from 2382.20 ± 314.39 mm^3^ to 2712.22 ± 343.55 mm^3^ and from 1350.22 ± 113.84 mm^3^ to 1596.10 ± 96.12 mm^3^ in MF (14% increase; *p* < 0.05) and MT (18% increase; *p* < 0.05), respectively. In general, we believe that the effect of cartilage repair on medial cartilage was better than that on lateral cartilage.

In the control group, no evidence of cartilage regeneration was found in patients with K-L grade 2 and 3 OA (Table 3). To make matters worse, we found that the medial cartilage was more vulnerable to damage. In patients with K-L grade 2, the thickness of cartilage defect increased from 1.63 ± 0.24 mm to 1.90 ± 0.23 mm in MF (17% increase; *p* = 0.001), more than LF and T. The volume of cartilage defect increased from 133.01 ± 35.21 mm^3^ to 154.45 ± 37.19 mm^3^ in MT (16% increase; *p* = 0.076), more than LT. Similar to the patients with K-L grade 2, the most severely damaged cartilage in K-L grade 3 remains in the medial cartilage. The volume of cartilage defect increased from 267.43 ± 73.34 mm^3^ to 306.14 ± 76.03 mm^3^ in MF (14% increase; *p* = 0.100) and from 187.72 ± 31.95 mm^3^ to 216.26 ± 37.27 mm^3^ in MT (15% increase; *p* = 0.007). These results were similar to the view of cartilage repair in the test group.

### 3.3. Clinical and Radiological Outcome

**Figure 6:**
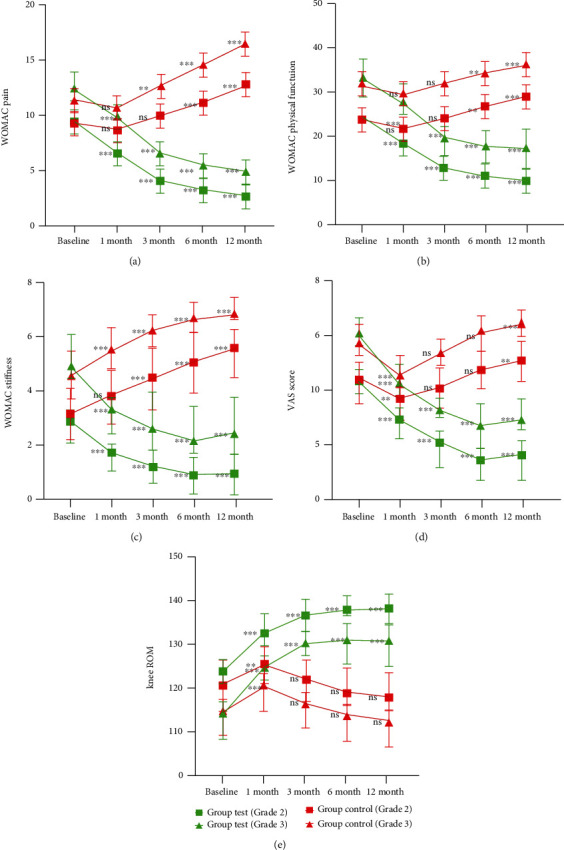
Changes of the VAS, ROM, WOMAC pain, stiffness, and physical function during 12-month follow-up after intra-articular injection of SVF and HA. Values in graphs are expressed as mean ± SD in vertical bars. ^∗∗^*p* < 0.01 and ^∗∗∗^*p* < 0.001. ns: nonsignificant (*p* > 0.05). All values were compared with baseline: (a) WOMAC pain; (b) WOMAC physical function; (c) WOMAC stiffness; (d) VAS score; (e) knee ROM.

After one year of follow-up, the VAS, WOMAC pain, stiffness, and physical function of the patients were evaluated at baseline and 1, 3, 6, and 12 months after injection with SVF and HA (Figure 6). In the test group, the mean WOMAC pain, stiffness, and physical function scores decreased from 9.38 ± 0.96 to 2.69 ± 1.02, from 2.83 ± 0.75 to 0.93 ± 0.74, and from 24.66 ± 3.12 to 10.14 ± 2.24 in the patients with grade 2 OA, while those scores of patients with grade 3 OA also showed a significant improvement. The mean VAS scores improved from 4.31 ± 0.46 to 1.59 ± 0.93 in grade 2 and from 6.04 ± 0.61 to 2.88 ± 0.78 in grade 3. In the control group, the mean WOMAC pain, stiffness, physical function, and VAS scores were relieved by one month after HA injection in grades 2 and 3 but were amplified again at 3-, 6-, and 12-month visits.

Functional improvement of ROM was significant at one month after HA therapy, from 120.59 ± 5.83° to 125.24 ± 4.15° in grade 2 and from 114.75 ± 5.54° to 120.46 ± 4.90° in grade 3. However, this trend took a turn for the worse after three months postoperation in the control group. Unlike the HA-treated group, the improvement of ROM showed a statistically significant difference, improving from 123.72 ± 3.44° to 137.82 ± 3.44° and from 114.21 ± 5.97° to 130.62 ± 5.72° in grade 2 and 3 OA, respectively.

The whole-organ assessment of the knees was performed by the WORMS at baseline and 6-month and 12-month follow-up (Table 4). In the test group, we subsequently found no signs of new cyst formation, neoplasms of the bone, cartilage, and synovium. The mean WORMS improved from a baseline of 54.86 ± 8.15 to 40.48 ± 7.28 at 12 months, in patients with grade 2 OA. Likewise, in grade 3 OA, the WORMS decreased from a baseline of 75.67 ± 10.44 to 57.46 ± 8.03, which revealed a significant improvement. By contrast, the consequence in the control group was poor; the WORMS deteriorated to 66.90 ± 11.15 and 84.04 ± 7.31 in patients with grade 2 and 3 OA, respectively.

The repair of the cartilage defects was measured using the MOCART system at 6 and 12 months (Table 5). As for the test group, the MOCART score improved from 52.93 ± 13.87 to 62.07 ± 12.83 at 6 and 12 months, respectively, in patients with K-L grade 2. Similarly, it increased from 46.46 ± 10.05 to 57.08 ± 11.98 at 6 and 12 months in patients with K-L grade 3, respectively. However, the MOCART score of the control group was decreased from 25.37 ± 12.40 to 17.71 ± 13.43 and from 22.41 ± 9.94 to 13.54 ± 6.34, at 6 and 12 months in grade 2 and 3 OA, respectively.

**Figure 7:**
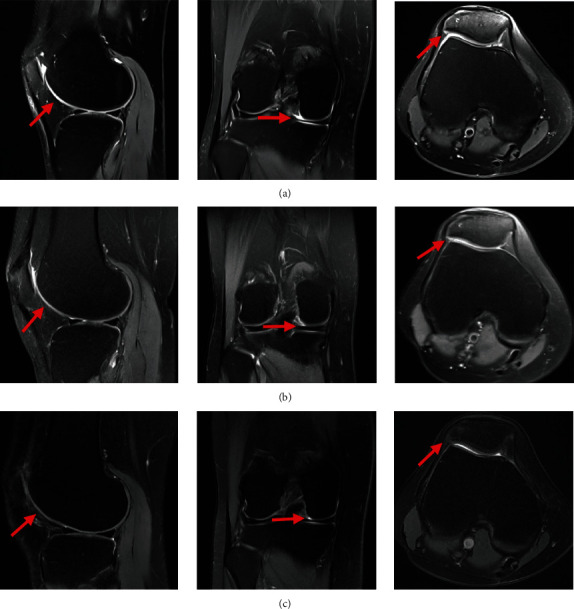
MRI scans of the SVF-treated knees with OA performed at baseline and 6 and 12 months, respectively. It was found that the defect was completely repaired and filled, and the cartilage fused well with adjacent cartilage and subchondral bone in the coronal, transverse, and sagittal planes (red arrow): (a) baseline; (b) 6 months; (c) 12 months.

In addition, there were 12 knees (41.38%) that showed complete or hypertrophic repair tissue filling of the defect in grade 2 OA, while 13 knees (44.83%) elucidated most of the repair of cartilage defects (Figure 7). Although only one knee (4.17%) showed complete repair of the cartilage defects in grade 3 OA, there were 18 knees (75.00%) that showed substantial repair of cartilage defects. In the control group, there were 5 knees (18.52%) that showed substantial repair of cartilage defects in grade 2, and only one knee (4.17%) showed substantial repair of cartilage defects in grade 3.

## 4. Discussion

Nonoperative therapy is a frequently prescribed option for knee osteoarthritis treatment. Unfortunately, conservative treatment has been found to only temporarily relieve clinical symptoms, while their long-term efficacy is not satisfactory, eventually requiring an alternative intervention, TKA. Previous studies have highlighted that TKA may be associated with life-threatening complications such as infection, thromboembolism, myocardial infarction, and even death. In addition, the life span of the prosthesis is between 10 and 15 years [27]. Therefore, it will be of great significance to find an effective treatment particularly for reversing the progression of this disease. Interestingly, numerous studies have recently confirmed that intra-articular injection of autologous adipose-derived SVF for the treatment of OA pain is safe and feasible [28–31]. However, most clinical studies on SVF had small sample sizes, so estimates from individual studies may be imprecise, and their radiological evaluation only remains at the 2D level. So far, it remains enigmatic whether SVF can promote the growth of cartilage.

Furthermore, multiple recent studies have reported inconsistent findings of the effect of SVF on cartilage regeneration. For instance, Hong et al. found that the knee joint exhibited significant defect filling and cartilage repair after receiving SVF. Similarly, WORMS and MOCART scores verified this conclusion [32]. Jo et al. used the parameters of the 3D cartilage model to verify the efficacy of cartilage repair but did not use the special MRI sequence; the appearance of the cartilage model was poor [31]. In 2017, Nguyen proposed that the cartilage regeneration of the knee joint after Arthroscopic Microfracture (AM) combined with SVF/PRP injection was probably due to the combination of SVF and platelet-rich plasma (PRP), where SVF is the primary factor of this healing reaction. Elsewhere, several studies confirmed that PRP significantly reduced short-term pain without cartilage regeneration [33, 34]. In a double-blinded prospective randomized controlled clinical trial, Garza et al. reported no significant difference in cartilage thickness between the test group injected with SVF and the control group injected with placebo. However, in this study, participants were followed for only six months [35]. In the final analysis, these studies had small sample sizes, so estimates from individual studies may be imprecise, and their radiological evaluation remains at the 2D level.

The emergence of the 3D-FS-SPGR sequence and finite element analysis provided a new method to evaluate cartilage quantitatively. Kijowski et al. proposed that routine MRI with a 3D sequence can improve the diagnostic performance for detecting cartilage lesions in the knee [36]. Jang et al. believed that the 3D-SPGR sequence can provide better diagnostic performance for the evaluation of knee articular cartilage lesions by detecting partial-thickness cartilage lesions in patients with OA [37]. In 2014, Peterfy et al. combined the 3D-SPGR sequence with finite element analysis to establish the cartilage model for accurate prediction of normal intra-articular pressure and force under different loads [25]. By finite element simulation, Li et al. proposed that meniscectomy can relieve pain for some time, resulting in more severe biomechanical changes and increase progression of cartilage injury [38]. Taken together, these studies confirmed that the 3D-SPGR sequence can provide better diagnostic performance, and the cartilage model is reliable. However, no scholar has applied this technology to evaluate cartilage regeneration of SVF.

Herein, we enrolled 95 patients with K-L grade 2 and 3 OA in this study. Each patient underwent the examination of the 3D-FS-SPGR sequence before treatment and at 6 and 12 months. We employed the 3D-FS-SPGR sequence to develop a 3D cartilage model, thereby dividing the cartilage at the 3D level. Compared with the conventional MRI sequence, the slice thickness of the 3D-FS-SPGR sequence was 1 mm, which can reduce the volume effect on imaging, and consequently, the resulting data is more accurate. In addition, the 3D SPGR sequence was clearer and more stratified for the imaging of articular cartilage, the interslice gaps of the 3D-SPGR sequence is 0 mm, and the error of the 3D modeling is less, which can be used for the quantitative analysis of cartilage. Following this, we recorded the changes of cartilage parameters in each region. Remarkably, the cartilage of all regions was improved to some extent in the test group, especially the MF and MT. In grade 2 OA, the thickness, volume, and size of cartilage defect in MF decreased to 0.92 ± 0.18 mm, 84.00 ± 32.30 mm^3^, and 182.22 ± 67.00 mm^2^, respectively. These parameters decreased to 0.96 ± 0.22 mm, 64.18 ± 21.40 mm^3^, and 146.15 ± 45.47 mm^2^ in MT. Similarly, these parameters of cartilage defects in MF and MT were greatly improved in grade 3, more than other regions. We identified that the efficacy of patients with medial cartilage injury was better compared with that of patients with other region injuries, whether pain improvement, functional recovery, or cartilage repair. We did not compare the MRI results with arthroscopy; the secondary surgery can cause injury to the patient, even though it is a minimally invasive procedure.

Besides, we further observed that the cartilage of patients with K-L grades 2 and 3 had different responses to SVF injection. The WOMAC score, ROM, and rehabilitation speed of patients with grade 2 were better than those of patients with grade 3. The WOMAC pain, stiffness, and physical function scores decreased from 9.38 ± 0.96 to 2.69 ± 1.02 (71% decrease), from 2.83 ± 0.75 to 0.93 ± 0.74 (67% decrease), and from 24.66 ± 3.12 to 10.14 ± 2.24 (59% decrease) in grade 2, while those scores in grade 3 improved to 4.92 ± 1.22 (60% decrease), 2.41 ± 1.35 (51% decrease), and 17.58 ± 4.35 (48% decrease), respectively. Likewise, based on the degree of cartilage repair, the increase in grade 2 OA was higher compared to that of grade 3 OA. There were 12 knees (41.38%) that showed complete or hypertrophic repair tissue filling of the defect in grade 2, and 13 knees (44.83%) elucidated most (beyond 50%) repair of cartilage defects. Only one knee (4.17%) showed complete repair of the cartilage defects in grade 3 OA. In summary, these results confirmed that SVF cell therapy can effectively improve clinical symptoms and promote cartilage regeneration before the excessive development of cartilage degeneration.

However, despite these promising results, this work has some limitations that are worth noting. First, the segmentation of the image was done through manual segmentation, which would increase some errors. Then, the follow-up period was short (12 months), whereby clinical evaluations were performed at baseline and 1, 3, 6, and 12 months after intra-articular injection of SVF cells into the knee. Third, we did not evaluate the relationship between the intra-articular injection dose of SVF cells and clinical results; hence, the effect of dose on clinical efficacy is not clear. Finally, although the MRI and parameters of the cartilage model clearly elucidated the regeneration of articular cartilage, it remains elusive whether the regenerated cartilage was either fibrocartilage or hyaline cartilage.

## 5. Conclusion

Collectively, our study demonstrates that autologous adipose-derived SVF can effectively relieve pain and improve function. We noted that the method of establishing the model and calculating parameters through the 3D-FS-SPGR sequence can accurately evaluate the effect of cartilage repair. Quantitative data of the cartilage model showed significant improvements in cartilage regeneration. Therefore, this research suggests that intra-articular injection of SVF is a promising minimally invasive therapy for cartilage regeneration, particularly for K-L grades 2 and 3.

## Figures and Tables

**Table 1:** Study participant demographic characteristics.

Characteristics	Test group (knee treated with SVF)	Control group (knee treated with HA)
Age (years)	50.83 ± 10.88	52.87 ± 9.35
Sex (M/F), *n* (%)	18/29 (38%/62%)	20/28 (42%/58%)
Knee (R/L), *n* (%)	30/23 (57%/43%)	21/30 (41%/59%)
BMI (kg/m^2^)	22.67 ± 3.68	23.58 ± 4.19
K-L classification (%)		
I	0 (0%)	0 (0%)
II	29 (55%)	27 (53%)
III	24 (45%)	24 (47%)
IV	0 (0%)	0 (0%)

**Table 2:** The changes of the cartilage model in the test group.

	Volume of defective cartilage (mm^3^)	*p* value	Size of defective cartilage (mm^2^)	*p* value	Volume of healthy cartilage (mm^3^)	*p* value	Thickness of defective cartilage (mm)	*p* value
Grade 2								
MF								
Baseline	173.82 ± 63.41		353.86 ± 122.99		3102.37 ± 435.02		1.53 ± 0.23	
6 months	123.13 ± 46.87	<0.001	257.17 ± 95.64	<0.001	3231.87 ± 451.13	0.279	1.16 ± 0.20	<0.001
12 months	84.00 ± 32.30	<0.001	182.22 ± 67.00	<0.001	3317.69 ± 447.02	0.073	0.92 ± 0.18	<0.001
LF								
Baseline	146.10 ± 61.17		302.77 ± 101.75		3070.04 ± 428.12		1.46 ± 0.30	
6 months	116.49 ± 51.34	<0.05	244.22 ± 96.33	<0.05	3116.65 ± 422.88	0.557	1.25 ± 0.27	<0.05
12 months	94.73 ± 45.55	<0.001	199.93 ± 86.07	<0.001	3179.09 ± 426.00	0.343	1.17 ± 0.26	<0.001
T								
Baseline	147.91 ± 61.35		309.72 ± 99.22		2568.48 ± 406.67		1.45 ± 0.25	
6 months	127.76 ± 57.33	0.318	262.86 ± 97.90	0.172	2617.60 ± 408.53	0.645	1.34 ± 0.23	0.153
12 months	112.80 ± 56.09	0.085	222.52 ± 98.57	<0.05	2658.51 ± 410.85	0.412	1.25 ± 0.21	<0.05
MT								
Baseline	139.72 ± 46.15		281.79 ± 80.48		1647.92 ± 200.24		1.43 ± 0.26	
6 months	95.43 ± 31.56	<0.001	206.20 ± 63.30	<0.001	1720.68 ± 197.61	0.178	1.15 ± 0.23	<0.001
12 months	64.18 ± 21.40	<0.001	146.15 ± 45.47	<0.001	1783.31 ± 202.94	<0.05	0.96 ± 0.22	<0.001
LT								
Baseline	119.87 ± 32.51		256.78 ± 64.51		1613.65 ± 147.04		1.34 ± 0.19	
6 months	101.62 ± 30.18	0.055	209.44 ± 56.13	<0.05	1656.77 ± 150.76	0.284	1.22 ± 0.19	<0.05
12 months	88.66 ± 28.04	<0.05	178.79 ± 54.55	<0.001	1694.24 ± 150.56	<0.05	1.13 ± 0.18	<0.001
P								
Baseline	137.29 ± 53.30		292.45 ± 106.74		2304.81 ± 181.21		1.29 ± 0.19	
6 months	117.78 ± 46.70	0.347	247.55 ± 89.12	0.268	2354.98 ± 182.95	0.304	1.10 ± 0.16	<0.05
12 months	102.15 ± 43.47	0.095	213.88 ± 82.64	0.057	2394.72 ± 180.11	0.067	1.01 ± 0.15	<0.001
Grade 3								
MF								
Baseline	278.10 ± 110.58		525.43 ± 167.38		2382.20 ± 314.39		1.72 ± 0.32	
6 months	198.80 ± 79.19	<0.05	408.84 ± 144.89	<0.05	2540.67 ± 323.21	0.105	1.34 ± 0.25	<0.001
12 months	130.30 ± 49.56	<0.001	286.18 ± 108.47	<0.001	2712.22 ± 343.55	<0.05	1.03 ± 0.23	<0.001
LF								
Baseline	229.23 ± 94.05		459.71 ± 176.88		2379.37 ± 235.44		1.74 ± 0.28	
6 months	190.17 ± 79.75	0.111	390.81 ± 153.97	0.144	2472.52 ± 270.39	0.241	1.53 ± 0.25	<0.05
12 months	162.17 ± 70.92	<0.05	339.47 ± 144.43	<0.05	2562.15 ± 276.73	<0.05	1.36 ± 0.23	<0.001
T								
Baseline	196.75 ± 77.85		410.31 ± 152.60		2190.18 ± 198.06		1.55 ± 0.30	
6 months	166.80 ± 69.83	0.179	352.21 ± 139.03	0.189	2261.72 ± 210.30	0.256	1.34 ± 0.28	<0.05
12 months	141.78 ± 59.94	<0.05	304.62 ± 121.47	<0.05	2323.74 ± 226.45	<0.05	1.19 ± 0.27	<0.001
MT								
Baseline	200.96 ± 48.48		410.59 ± 88.53		1350.22 ± 113.84		1.62 ± 0.21	
6 months	135.99 ± 26.49	<0.001	290.12 ± 51.28	<0.001	1477.44 ± 94.51	<0.001	1.27 ± 0.19	<0.001
12 months	95.11 ± 19.93	<0.001	208.12 ± 42.70	<0.001	1596.10 ± 96.12	<0.001	0.99 ± 0.14	<0.001
LT								
Baseline	154.40 ± 48.17		333.83 ± 98.97		1384.14 ± 92.13		1.47 ± 0.27	
6 months	131.21 ± 44.61	0.087	283.62 ± 89.28	0.070	1438.02 ± 94.16	0.058	1.31 ± 0.24	0.030
12 months	110.57 ± 39.86	<0.05	238.78 ± 81.67	<0.001	1473.00 ± 97.45	<0.05	1.16 ± 0.23	<0.001
P								
Baseline	140.84 ± 56.97		320.57 ± 112.90		1686.92 ± 117.79		1.41 ± 0.20	
6 months	117.97 ± 49.49	0.126	250.71 ± 100.31	0.083	1771.54 ± 112.93	0.016	1.23 ± 0.19	<0.05
12 months	98.75 ± 42.84	<0.05	209.57 ± 84.85	<0.05	1847.87 ± 117.22	<0.001	1.09 ± 0.19	<0.001

**Table 3:** The changes of the cartilage model in the control group.

	Volume of defective cartilage (mm^3^)	*p* value	Size of defective cartilage (mm^2^)	*p* value	Volume of healthy cartilage (mm^3^)	*p* value	Thickness of defective cartilage (mm)	*p* value
Grade 2								
MF								
Baseline	183.82 ± 48.24		356.83 ± 91.08		3164.07 ± 411.84		1.63 ± 0.24	
6 months	197.64 ± 48.89	0.374	382.42 ± 92.72	0.384	3139.72 ± 412.82	0.829	1.78 ± 0.24	0.04
12 months	209.02 ± 48.30	0.114	402.29 ± 91.64	0.124	3107.26 ± 413.46	0.615	1.90 ± 0.23	0.001
LF								
Baseline	140.82 ± 43.70		275.08 ± 84.19		3077.84 ± 431.44		1.52 ± 0.29	
6 months	148.13 ± 42.87	0.545	291.72 ± 82.19	0.474	3067.12 ± 426.18	0.927	1.57 ± 0.32	0.588
12 months	154.00 ± 43.56	0.281	308.61 ± 82.01	0.152	3044.24 ± 430.33	0.776	1.60 ± 0.33	0.361
T								
Baseline	137.32 ± 59.12		279.60 ± 121.65		2607.93 ± 504.48		1.37 ± 0.21	
6 months	151.97 ± 63.42	0.318	298.40 ± 122.40	0.676	2589.15 ± 500.17	0.891	1.40 ± 0.22	0.518
12 months	165.57 ± 66.04	0.085	308.89 ± 119.60	0.512	2573.91 ± 501.18	0.805	1.44 ± 0.22	0.227
MT								
Baseline	133.01 ± 35.21		257.93 ± 59.75		1680.74 ± 196.00		1.49 ± 0.40	
6 months	142.19 ± 33.79	0.418	287.87 ± 58.95	0.129	1650.56 ± 190.03	0.568	1.58 ± 0.40	0.509
12 months	154.45 ± 37.19	0.076	318.45 ± 58.71	0.003	1618.74 ± 193.26	0.247	1.64 ± 0.41	0.272
LT								
Baseline	129.20 ± 38.74		255.47 ± 74.88		1672.37 ± 192.72		1.39 ± 0.27	
6 months	137.11 ± 39.48	0.553	270.05 ± 76.14	0.566	1651.50 ± 193.01	0.693	1.43 ± 0.27	0.603
12 months	142.37 ± 39.00	0.320	286.76 ± 74.68	0.218	1629.21 ± 188.67	0.409	1.46 ± 0.28	0.399
P								
Baseline	139.49 ± 36.09		277.21 ± 61.16		2332.80 ± 220.41		1.30 ± 0.17	
6 months	148.49 ± 36.94	0.589	293.81 ± 64.78	0.572	2307.06 ± 221.86	0.671	1.35 ± 0.16	0.463
12 months	158.32 ± 37.93	0.270	307.47 ± 62.35	0.299	2286.39 ± 219.81	0.442	1.40 ± 0.17	0.169
Grade 3								
MF								
Baseline	267.43 ± 73.34		480.77 ± 131.81		2351.03 ± 235.53		1.60 ± 0.37	
6 months	286.20 ± 77.66	0.406	512.16 ± 135.12	0.486	2317.02 ± 239.61	0.622	1.70 ± 0.36	0.401
12 months	306.14 ± 76.03	0.100	542.38 ± 136.31	0.177	2291.33 ± 241.71	0.391	1.77 ± 0.35	0.123
LF								
Baseline	240.85 ± 96.23		477.24 ± 187.46		2421.01 ± 324.67		1.73 ± 0.26	
6 months	256.56 ± 97.23	0.629	503.78 ± 187.78	0.674	2388.35 ± 318.51	0.727	1.82 ± 0.26	0.300
12 months	264.44 ± 105.07	0.788	530.49 ± 189.86	0.710	2363.33 ± 322.17	0.540	1.90 ± 0.25	0.109
T								
Baseline	214.74 ± 75.26		421.14 ± 148.53		2289.15 ± 308.65		1.51 ± 0.37	
6 months	233.95 ± 77.94	0.529	451.43 ± 145.69	0.604	2247.38 ± 310.62	0.637	1.62 ± 0.37	0.459
12 months	251.24 ± 80.86	0.245	478.47 ± 147.10	0.333	2220.16 ± 306.64	0.401	1.71 ± 0.36	0.172
MT								
Baseline	187.72 ± 31.95		368.70 ± 65.61		1368.12 ± 91.07		1.56 ± 0.33	
6 months	202.21 ± 29.26	0.108	387.01 ± 60.22	0.319	1349.76 ± 101.26	0.512	1.63 ± 0.31	0.459
12 months	216.26 ± 37.27	0.007	412.67 ± 75.35	0.036	1335.96 ± 108.87	0.273	1.68 ± 0.33	0.241
LT								
Baseline	152.32 ± 50.11		306.34 ± 87.99		1363.31 ± 117.82		1.54 ± 0.39	
6 months	168.41 ± 51.88	0.448	324.18 ± 92.46	0.633	1336.30 ± 121.08	0.438	1.63 ± 0.38	0.598
12 months	182.72 ± 54.90	0.171	338.17 ± 108.00	0.437	1312.25 ± 109.98	0.128	1.69 ± 0.38	0.368
P								
Baseline	160.01 ± 58.53		302.71 ± 106.39		1626.33 ± 154.17		1.55 ± 0.29	
6 months	174.43 ± 61.54	0.563	330.44 ± 107.92	0.533	1599.34 ± 149.72	0.541	1.61 ± 0.29	0.588
12 months	186.20 ± 63.81	0.306	354.39 ± 113.11	0.261	1564.71 ± 155.23	0.174	1.69 ± 0.30	0.253

**Table 4:** WORMS changes during 12-month follow-up.

Variables	Grade 2					Grade 3				
	Baseline	6 months	*p* value	12 months	*p* value	Baseline	6 months	*p* value	12 months	*p* value
Test group										
Cartilage	26.48 ± 3.43	19.38 ± 2.91	<0.001	15.17 ± 2.96	<0.001	34.33 ± 5.89	25.75 ± 4.39	<0.001	19.58 ± 3.83	<0.001
Marrow abnormality	3.07 ± 1.01	1.97 ± 0.96	<0.001	1.72 ± 0.91	<0.001	4.42 ± 1.11	3.25 ± 0.88	<0.001	2.88 ± 0.60	<0.001
Bone cysts	2.31 ± 1.02	1.76 ± 0.94	<0.05	1.69 ± 0.91	<0.05	3.71 ± 0.68	3.04 ± 0.68	<0.05	2.91 ± 0.70	<0.001
Bone attrition	1.03 ± 0.93	0.90 ± 0.80	0.535	0.83 ± 0.75	0.353	2.50 ± 0.65	2.38 ± 0.63	0.500	2.25 ± 0.60	0.179
Osteophytes	19.97 ± 3.99	19.69 ± 4.07	0.799	19.59 ± 4.03	0.726	27.08 ± 4.75	26.67 ± 4.76	0.766	26.63 ± 4.68	0.743
Menisci	0.83 ± 1.12	0.59 ± 0.81	0.325	0.55 ± 0.77	0.261	1.67 ± 1.28	1.50 ± 1.08	0.628	1.54 ± 1.18	0.716
Ligaments	0.07 ± 0.25	0.03 ± 0.18	0.538	0.03 ± 0.18	0.538	0.17 ± 0.37	0.13 ± 0.33	0.669	0.08 ± 0.28	0.393
Synovitis	1.10 ± 0.71	0.93 ± 0.74	0.374	0.90 ± 0.71	0.286	1.79 ± 0.64	1.63 ± 0.75	0.436	1.58 ± 0.76	0.330
WORMS total	54.86 ± 8.15	45.24 ± 7.52	<0.001	40.48 ± 7.28	<0.001	75.67 ± 10.44	64.33 ± 9.09	<0.001	57.46 ± 8.03	<0.001
Control group										
Cartilage	26.41 ± 4.48	28.59 ± 4.73	0.078	30.48 ± 4.82	0.002	34.08 ± 5.12	35.96 ± 4.39	0.18	37.17 ± 3.18	0.017
Marrow abnormality	3.48 ± 1.35	6.31 ± 2.16	<0.001	6.76 ± 1.57	<0.001	4.46 ± 1.25	7.54 ± 0.83	<0.001	7.67 ± 0.64	<0.001
Bone cysts	2.13 ± 1.19	2.38 ± 1.01	0.409	2.89 ± 0.71	0.036	3.33 ± 0.76	3.67 ± 0.48	0.078	3.79 ± 0.41	0.014
Bone attrition	1.38 ± 0.86	3.17 ± 1.34	<0.001	3.38 ± 1.01	<0.001	2.21 ± 0.78	4.79 ± 1.14	<0.001	4.96 ± 1.08	<0.001
Osteophytes	20.21 ± 4.90	20.59 ± 5.15	0.775	20.76 ± 4.87	0.669	26.29 ± 5.86	26.58 ± 5.56	0.86	26.83 ± 5.28	0.738
Menisci	0.97 ± 0.94	1.10 ± 0.94	0.579	1.24 ± 0.87	0.253	1.42 ± 0.72	1.54 ± 0.66	0.532	1.67 ± 0.56	0.186
Ligaments	0.14 ± 0.35	0.17 ± 0.38	0.723	0.24 ± 0.44	0.324	0.29 ± 0.46	0.33 ± 0.48	0.762	0.42 ± 0.50	0.376
Synovitis	0.97 ± 0.73	1.21 ± 0.73	0.212	1.34 ± 0.61	0.037	1.25 ± 0.68	1.46 ± 0.59	0.260	1.54 ± 0.51	0.098
WORMS total	55.69 ± 10.25	63.52 ± 11.79	0.009	66.90 ± 11.15	<0.001	73.33 ± 9.92	81.88 ± 8.19	0.002	84.04 ± 7.31	<0.001

**Table 5:** MOCART changes during 12-month follow-up.

Variables	Maximum score	Group test (grade 2/3), *n* (%)		Group control (grade 2/3), *n* (%)	
		6 months	12 months	6 months	12 months
1. Degree of defect repair and filling of the defect					
Complete	20	0 (0)/0 (0)	2 (6.90)/1 (4.17)	0 (0)/0 (0)	0 (0)/0 (0)
Hypertrophy	15	9 (31.03)/5 (20.83)	10 (34.48)/8 (33.33)	2 (7.41)/0 (0)	2 (7.41)/0 (0)
Incomplete					
>50% of the adjacent cartilage	10	12 (41.38)/9 (37.50)	13 (44.83)/10 (41.66)	4 (14.81)/2 (8.33)	3 (11.11)/1 (4.17)
<50% of the adjacent cartilage	5	7 (21.14)/7 (29.17)	4 (13.79)/4 (16.67)	11 (40.74)/7 (29.17)	11 (40.74)/6 (25.00)
Subchondral bone exposed	0	1 (3.45)/3 (12.50)	0 (0)/1 (4.17)	10 (37.04)/15 (62.50)	11 (40.74)/17 (70.83)
2. Integration to the border zone					
Complete	15	7 (21.14)/4 (16.67)	14 (48.28)/7 (29.16)	0 (0)/0 (0)	0 (0)/0 (0)
Incomplete					
Demarcating border visible (split-like)	10	17 (58.62)/10 (41.67)	12 (41.38)/9 (37.50)	5 (18.52)/3 (12.50)	4 (14.81)/3 (12.50)
Defect visible					
<50% of length of the repair tissue	5	3 (10.34)/7 (29.17)	3 (10.34)/6 (25.00)	11 (40.74)/7 (29.17)	12 (44.45)/6 (25.00)
>50% of length of the repair tissue	0	2 (6.90)/3 (12.50)	0 (0)/2 (8.33)	11 (40.74)/14 (58.33)	11 (40.74)/15 (62.50)
3. Surface of the repair tissue					
Surface intact	10	16 (55.17)/13 (54.17)	19 (65.52)/16 (66.67)	1 (3.70)/0 (0)	1 (3.70)/0 (0)
Surface damaged					
<50% of repair tissue depth	5	11 (37.93)/9 (37.50)	10 (34.48)/7 (29.16)	14 (51.85)/8 (33.33)	12 (44.45)/7 (29.17)
>50% of repair tissue depth or total degeneration	0	2 (6.90)/2 (8.33)	0 (0)/1 (4.17)	12 (44.45)/16 (66.67)	14 (51.85)/17 (70.83)
4. Structure of the repair tissue					
Homogeneous	5	20 (68.97)/16 (66.67)	23 (79.31)/19 (79.17)	9 (33.33)/5 (20.83)	8 (29.63)/7 (29.17)
Inhomogeneous or cleft formation	0	9 (31.03)/8 (33.33)	6 (20.69)/5 (20.83)	18 (66.67)/19 (79.17)	19 (70.37)/17 (70.83)
5. Signal intensity of repair tissue					
Normal (identical to adjacent cartilage)	30	2 (6.90)/1 (4.17)	7 (24.14)/3 (12.50)	2 (7.41)/1 (4.17)	2 (7.41)/0 (0)
Nearly normal (slight areas of signal alteration)	15	20 (68.96)/16 (66.67)	18 (62.07)/17 (70.83)	7 (25.92)/5 (20.83)	6 (22.22)/5 (20.83)
Abnormal (large areas of signal alteration)	0	7 (21.14)/7 (29.16)	4 (13.79)/4 (16.67)	18 (66.67)/18 (75.00)	19 (70.37)/19 (79.17)
6. Subchondral lamina					
Intact	5	21 (72.41)/17 (70.83)	21 (72.41)/20 (83.33)	12 (44.44)/7 (29.17)	7 (37.04)/4 (16.67)
Not intact	0	8 (27.59)/7 (29.17)	8 (27.59)/4 (16.67)	15 (55.56)/17 (70.83)	17 (62.96)/20 (83.33)
7. Subchondral bone					
Intact	5	8 (27.59)/8 (33.33)	8 (27.59)/11 (45.83)	6 (22.22)/6 (25.00)	4 (14.81)/4 (16.67)
Not intact (edema, granulation tissue, cysts, sclerosis)	0	21 (72.41)/16 (66.67)	21 (72.41)/13 (54.17)	21 (77.78)/18(75.00)	23 (85.19)/20 (83.33)
8. Adhesions					
No	5	18 (62.07)/12 (50.00)	18 (62.07)/16 (66.67)	8 (29.63)/7 (29.17)	6 (22.22)/6 (25.00)
Yes	0	11 (37.93)/12 (50.00)	11 (37.93)/8 (33.33)	19 (70.37)/17 (70.83)	21 (77.78)/18 (75.00)
9. Synovitis					
No synovitis	5	9 (31.03)/2 (8.33)	9 (31.03)/4 (16.67)	8 (29.63)/7 (29.17)	6 (22.22)/6 (25.00)
Synovitis	0	20 (68.97)/22 (91.67)	20 (68.97)/20 (83.33)	19 (70.37)/17 (70.83)	21 (77.78)/18 (75.00)
Mean ± SD		52.93 ± 13.87/46.46 ± 10.05	62.07 ± 12.83/57.08 ± 11.98	25.37 ± 12.40/17.71 ± 13.43	22.41 ± 9.94/13.54 ± 6.34

## Data Availability

The datasets generated and analyzed during the current study are available from the corresponding author on reasonable request.
